# Non-Genomic Actions of the Androgen Receptor in Prostate Cancer

**DOI:** 10.3389/fendo.2017.00002

**Published:** 2017-01-17

**Authors:** Jacky K. Leung, Marianne D. Sadar

**Affiliations:** ^1^Department of Genome Sciences Centre, British Columbia Cancer Agency, Vancouver, BC, Canada

**Keywords:** androgen receptor, non-genomic signaling, prostate cancer, AR antagonists, Src kinase, MAPK/ERK signaling, PI3K/Akt signaling

## Abstract

Androgen receptor (AR) is a validated drug target for prostate cancer based on its role in proliferation, survival, and metastases of prostate cancer cells. Unfortunately, despite recent improvements to androgen deprivation therapy and the advent of better antiandrogens with a superior affinity for the AR ligand-binding domain (LBD), most patients with recurrent disease will eventually develop lethal metastatic castration-resistant prostate cancer (CRPC). Expression of constitutively active AR splice variants that lack the LBD contribute toward therapeutic resistance by bypassing androgen blockade and antiandrogens. In the canonical pathway, binding of androgen to AR LBD triggers the release of AR from molecular chaperones which enable conformational changes and protein–protein interactions to facilitate its nuclear translocation where it regulates the expression of target genes. However, preceding AR function in the nucleus, initial binding of androgen to AR LBD in the cytoplasm may already initiate signal transduction pathways to modulate cellular proliferation and migration. In this article, we review the significance of signal transduction pathways activated by rapid, non-genomic signaling of the AR during the progression to metastatic CRPC and put into perspective the implications for current and novel therapies that target different domains of AR.

## Castration-Resistant Prostate Cancer

Approximately 30% of patients will relapse after primary therapy (1). Androgen deprivation therapy, both surgical and biochemical castration, is the main treatment for relapsed patients and provides temporary relief to tumor burden; however, all patients will acquire lethal, castration-resistant prostate cancer (CRPC). Androgen receptor (AR)-targeted therapies for blocking the androgen signaling axis have improved, with more potent, second-generation antiandrogens such as enzalutamide, and 17-hydroxylase/17,20-lyase (CYP17) inhibitors, but these agents only increase median overall survival by approximately 4 months in chemotherapy-naïve patients (2, 3).

Most CRPC continues to depend on AR despite continued androgen deprivation therapy. This is apparent from a rising titer of serum prostate-specific antigen, which indicates biochemical failure and the transition of CRPC. Proposed mechanisms of continued AR activity throughout CRPC include upregulation of AR by amplification of the *AR* gene or overexpression of AR protein, synthesis of intratumoral androgens, stimulation of ligand-independent AR activity by epidermal growth factor (EGF) or interleukin-6 (IL-6) or by the mitogen-activated protein kinase (MAPK) cascade, phosphoinositide 3-kinase (PI3K)/Akt, and protein kinase A pathways, and the expression of constitutively active AR splice variants such as ARv567es and V7 (4–11).

Androgen receptor belongs to the nuclear receptor superfamily of proteins, along with the glucocorticoid receptor, progesterone receptor, mineralocorticoid, and estrogen receptors, which all share a modular structure composed of an unstructured N-terminal domain (NTD), a DNA-binding domain (DBD), and a C-terminal ligand-binding domain (LBD). These domains operate as individual folding units that contribute to the transformation of a transcriptionally active receptor. Unlike the LBD and DBD which are structured domains, the AR NTD is intrinsically disordered and mediates protein–protein interactions that are required for transactivation. During AR transactivation, the activation function 1 region (AF1) of the AR NTD acquires transient folding structures to bind transcriptional coactivators to bridge the AR to basal transcriptional machinery. Classic deletion analyses of the AR NTD identified two transcriptional activation units within AF1 (Tau1 and Tau5), which represent surfaces that mediate ligand-dependent and ligand-independent transactivation, respectively (12, 13).

**Figure 1:**
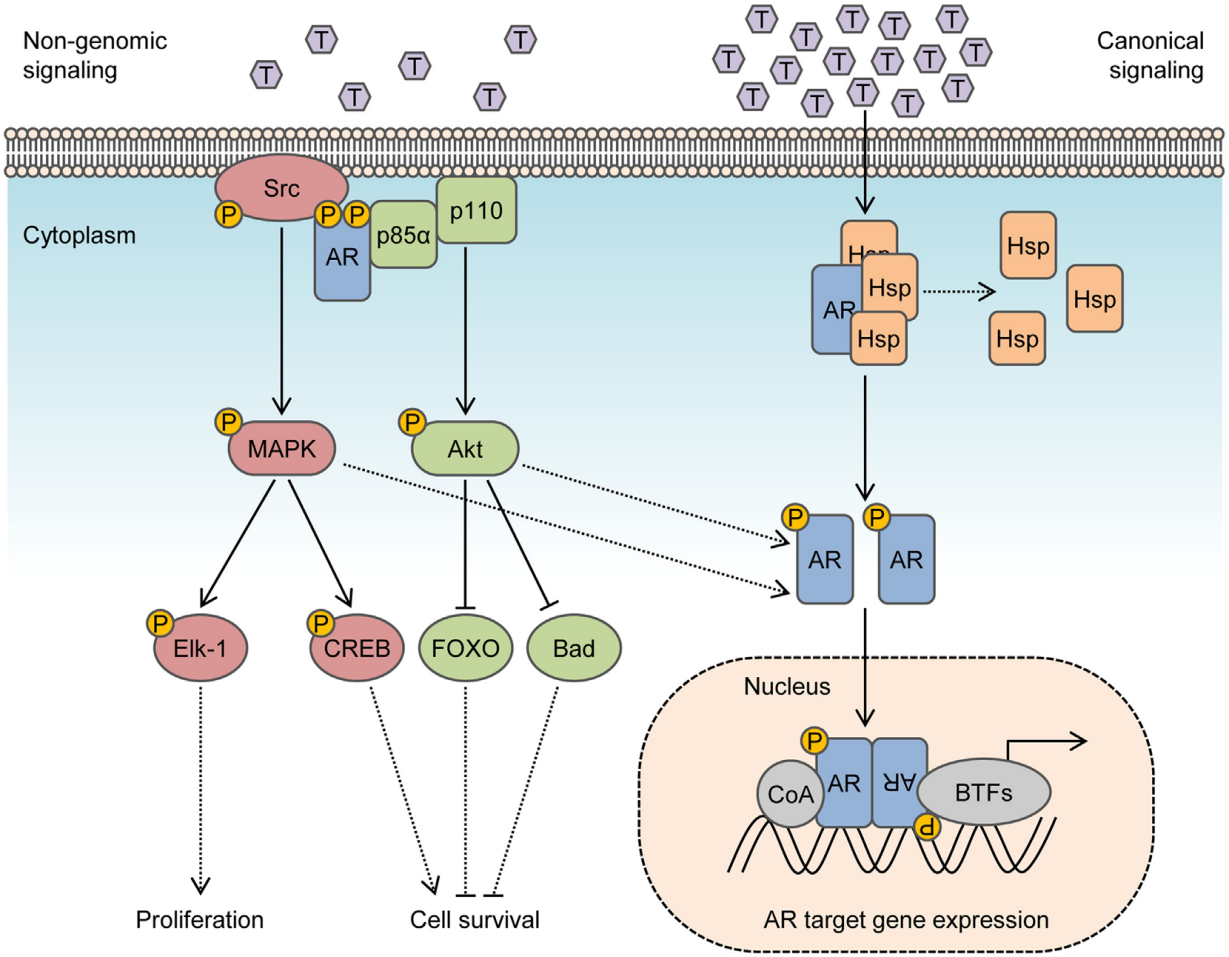
**Integration of non-genomic signaling and canonical signaling of androgen receptor (AR)**. In the presence of low androgen levels (picomolar concentrations), AR interactions with Src kinase and p85α regulatory subunit of phosphoinositide 3-kinase activates mitogen-activated protein kinase (MAPK) and Akt pathways to enhance cell proliferation and survival in a non-genomic fashion. In the presence of high androgen levels (nanomolar concentrations), AR is activated in a canonical pathway to regulate the expression of target genes. Activation of MAPK and Akt by non-genomic signaling also enhances genomic AR signals by phosphorylating the AR or transcriptional coactivators.

In the canonical model, inactive AR is maintained in the cytosol by molecular chaperones that include heat shock proteins, co-chaperones, and cytoskeletal proteins (14). Binding of AR to its natural ligand dihydrotestosterone (DHT) or testosterone triggers dissociation of chaperones and induces conformational changes that enable AR dimerization and interactions with a cytoskeletal protein, Filamin A, to modulate nuclear translocation and target gene expression (15, 16). Even so, many of the cellular responses to androgen do not fit in the canonical model and do not require transcription mediated by AR. This is because ligand-transformed AR is able to associate with molecular substrates in the cytoplasm and inner leaflet of the cell membrane to activate intracellular kinase cascades. These actions are referred to as rapid, non-genomic signaling of AR and enhance cell proliferation and survival by rapid signal transduction (Figure 1). In contrast to altering gene expression and synthesizing new proteins which may take hours, non-genomic actions of AR precede transcriptional events and are generally observed within minutes after exposure to androgen. Herein, we review prominent intracellular signaling pathways activated by non-genomic AR signaling in prostate cells and offer perspective into their implications for therapies targeting the AR.

## Non-Genomic AR Signaling

### Activation of Src Kinase

In response to androgen, a rapid association of AR with the non-receptor tyrosine kinase Src is responsible for enhancing cell proliferation through activation of the MAPK/ERK cascade. AR interacts with Src by binding of a polyproline sequence between residues 371 and 381 of the AR NTD to Src homology domain 3 (SH3). This association facilitates unfolding of Src and autophosphorylation to activate the Src kinase domain (17, 18). The importance of this interaction is emphasized in studies where deleting the polyproline sequence on AR or expressing peptides mimicking the polyproline region inhibits the activation of Src/ERK by AR and blocks the induction of human prostate or mammary cancer cell (LNCaP or MCF-7) growth by androgen (17, 19). Src/ERK induction is dependent on androgen concentration and is active in low to physiological androgen levels (0.1–10 nM) and inhibited by higher concentrations (100 nM) (20). This mirrors the biphasic effect of androgen, where low levels of androgens (0.01–0.1 nM) promote and high levels of androgens (1–100 nM) inhibit the growth of prostate cells (21–23). The biphasic effect of androgen to activate Src and stimulate proliferation is also observed in NIH3T3 fibroblast cells expressing low levels of AR, absent of AR nuclear translocation or transcription mediated by AR (24). These findings suggest that inhibitory effects on proliferation at high androgen levels come from a loss of transient AR protein–protein interactions with Src upon saturation of AR with ligand, rather than a diversion of AR to perform genomic functions. In NIH3T3 fibroblast cells, exposure to optimal concentration of androgen (10 nM) suppresses cell cycle progression, induces interactions between AR with the cytoskeletal protein Filamin A, and recruits integrin beta 1 to coordinate cell migration by activating focal adhesion kinase, paxillin, and Rac (25, 26). In malignant prostate tissue, cytoplasmic localization of Filamin A correlates with metastatic potential and an androgen-independent phenotype (27). Accordingly, forced nuclear localization of Filamin A may terminate non-genomic signals from AR supporting proliferation and restore bicalutamide sensitivity in C4-2 human prostate cancer cells, which exhibit androgen-independent growth (28).

Activation of the Ras-Raf-MAPK/ERK cascade is the primary mitogenic stimulus initiated by non-genomic AR effects observed in androgen-sensitive prostate cells. Levels of phospho-(p-) ERK1/2 peak within 5–30 min of exposure to DHT in LNCaP cells, PC-3 cells stably expressing wild-type AR, and primary prostatic stromal cells, but this is not detected in primary human genital skin fibroblasts until 16 h, implying cell-type specificity (20, 29). Activated ERK1/2 in response to androgen influences the activity of transcription factors in the nucleus that are independent of AR DNA binding, which in turn activates ETS domain-containing protein Elk-1 to regulate transcription of immediate early genes, including c-fos (29–31). Interestingly, antiandrogens (bicalutamide and flutamide) promoted the induction of p-ERK1/2 and induced transactivation of c-fos in reporter gene assays in PC3 cells only when wild-type AR was ectopically expressed (29). Moreover, p-ERK1/2 can also promote cell survival in a non-genomic manner by activating cAMP response element-binding protein (CREB) (20, 32). In a distinct manner, expression of a dominant negative CREB mutant in LNCaP cells abrogates DHT-induced protection against apoptosis, but does not prevent S-phase entry (20).

Aberrant Src activity is detected in malignant prostate cells and present in several AR-positive prostate cancer cell line models exhibiting androgen-independent growth (33–35). In low passage androgen-sensitive LNCaP cells, the ability for AR to activate Src and stimulate proliferation non-genomically is androgen dependent. However, in high passage LNCaP cells (more than 60 passages), AR interacts with Src constitutively and independently of androgen to promote growth under androgen-depleted conditions (20). In high passage LNCaP cells, the Src/MAP/ERK-1/2/CREB pathway is constitutively active, and only the MAPK inhibitor (PD98059) and not bicalutamide inhibits proliferation (20). Furthermore, while LNCaP cells do not typically form tumors in castrated hosts, high passage LNCaP cells do form tumors efficiently in castrated mice. C4-2 cells, a CRPC subline of LNCaP that can grow in castrated hosts, exhibit a threefold increase in protein expression of Src compared to androgen-sensitive LNCaP cells (36). Treatment of C4-2 cells with a Src inhibitor (PP2) inhibits growth, decreases invasive potential, and induces apoptosis, with synergy in combination with bicalutamide when bicalutamide alone has no inhibitory effects on this cell line (36). Supporting the role of Src in the progression of prostate cancer, immunohistochemistry of prostate tissue from the transgenic adenocarcinoma mouse prostate model shows a progressive increase (up to threefold) in positive staining of activated Src, as a function of age and cancer progression from 8 to 24 weeks (36). Since activating Src mutations are rare in human cancers, aberrant Src activity is presumably dependent on increased Src expression or stimulation by growth factors and interleukins abundant in the tumor microenvironment, including EGF, IGF, IL-6, IL-8 (37). Numerous studies provide evidence that Src inhibitors are effective in reducing proliferation and invasion of prostate cancer cell lines *in vitro* and demonstrate favorable antitumor activity *in vivo* using prostate cancer xenografts (38–40).

### Cross Talk with the PI3K Pathway

Activation of the PI3K/Akt pathway is also triggered by non-genomic AR signaling. Direct interactions between ligand-activated AR and PI3K in the cytosol are mediated by binding of phosphotyrosine residues on the AR NTD to SH2 domain of p85α regulatory subunit of PI3K (41). Association of AR/p85α promotes activation of p110 catalytic subunit and generation of phosphatidylinositol-3,4,5-trisphosphate (PIP_3_) signaling lipids to induce activation of Akt kinase, which leads to regulation of transcription factors to inhibit apoptosis pathways and promote cell survival. Accumulation of Akt p-S473 is detected over 10–30 min upon androgen stimulation of non-tumoral VDEC cells or PC3 cells when stably expressing AR, supporting its role as a non-genomic AR stimulus (41). Activated Akt phosphorylates proapoptotic protein BAD and forkhead box FOXO proteins to maintain cell survival (41). Among Akt substrates, FOXO3a enhances AR expression by direct binding to the *AR* promoter (42), whereas FOXO1 may decrease AR transactivation by recruitment of histone deacetylase HDAC3 (43). Collectively, these emphasize the role of AR in maintaining cell survival independently of transcription, which is enhanced by PI3K/Akt signaling. Negative regulation of PI3K/Akt is facilitated by conversion of PIP_3_ to PIP_2_ by phosphatase and tensin homolog (PTEN), which is a commonly lost tumor suppressor in prostate cancer. Indeed, genomic alterations affecting the PI3K/Akt pathway are detected in 42% of primary prostate tumors and 100% of metastatic tumors (44). Furthermore, Akt may also bind to and phosphorylate residues S213 and S791 on AR to modulate transactivation (10). Interestingly, Akt represses AR activity in low passage LNCaP cells, but promotes AR transcription in high passage LNCaP cells (>60 passages) where the PI3K/Akt pathway is aberrantly active (8).

Phosphotryosine residues on the AR NTD mediate the interaction between AR and PI3K, which include Y267, Y363, and Y534 (45). These residues are phosphorylated in response to EGF by Cdc42-associated kinase Ack on Y267 and Y363, or Src on Y534 (46, 47). Src-mediated phosphorylation of AR Y534 enhances the stability of AR by preventing interactions with E3 ligase CHIP that promote proteasomal degradation (48). Mutation of AR Y534 to phenylalanine disrupts the ability for AR to localize in the nucleus in a ligand-independent manner and inhibits AR transactivation in reporter gene assays in response to EGF or low concentrations of androgen (47). Studies in fibroblast cells support a ternary complex of Src/AR/p85α as a non-genomic AR signal (24). It is likely that an AR/Src complex is formed first by rapid signaling, followed by phosphorylation of AR Y534 by Src to recruit the p85α regulatory subunit of PI3K. Thus, non-genomic activation of AR and interactions between Src/AR and p85α can trigger concurrent activation of the MAPK and PI3K/Akt pathways to enhance cell proliferation and cell survival under androgen-depleted conditions.

### Convergence with Ligand-Independent Activation of AR

Our lab identified that the AR NTD can be activated independently of androgen, by intracellular kinase signals, cytokines, and osteoblast-derived factors (6, 7, 49). Ligand-independent activation of AR may be enhanced by the pathways activated in non-genomic AR signaling. Specifically, MAPK/ERK activation in response to IL-6 increases transactivation of the AR NTD through interactions with STAT3 (7). IL-8 stimulates androgen-independent growth and transactivation of AR in a Src and ERK-dependent manner (50). EGF signaling enhances ligand-independent AR transactivation and promotes activation of Src and the Ras-Raf-MAPK/ERK pathway (9, 51, 52). Notably, activated Src is also able to stimulate the STAT pathway and enhance signals by growth factor kinases, such as EGFR (53, 54). Moreover, activation of the MAPK/ERK and PI3K/Akt kinase pathways non-genomically can enhance ligand-independent transactivation of AR by modulating interactions with coactivators including steroid receptor coactivator-1 and androgen receptor-associated protein 70 (55, 56).

## Implications for Prostate Cancer Therapies

### Current AR Therapies

Non-genomic AR signaling pathways may likely contribute to resistance to androgen deprivation therapy and antiandrogens. Prostate cancer patients undergoing maximal androgen deprivation therapy with surgical orchiectomy or LHRH analog combined with an antiandrogen (bicalutamide or flutamide) still have low levels of testosterone in their serum (less than 0.1 nM), which is within the range of AR to mediate non-genomic responses (57, 58). Levels of androgen in CRPC tissue are at least 10-fold higher, in the 1–3 nM range (59). Bicalutamide and flutamide are not effective in blocking non-genomic activation of AR and show an inherent ability to induce p-ERK1/2 and c-fos by acting through AR in androgen-depleted conditions (29). More potent second-generation antiandrogens, such as enzalutamide, apalutamide (ARN-509), and darolutamide (ODM-201), have greater affinity for the AR LBD and may reduce nuclear translocation of full-length AR, resulting in accumulation AR in the cytosol (60–62). In the cytosol, the AR NTD has the potential to activate Src and PI3K, to drive tumor growth and survival. Concentrating AR in the cytoplasm may also effectively lower the androgen requirements for optimal growth, or work in concert with Src phosphorylation of AR Y534 to sensitize AR to low androgen concentrations. Furthermore, a consequence of AR inhibition is reduced expression of FKBP5, an AR target gene that is a chaperone for the phosphatase PHLPP targeting p-Akt. Accordingly, enhanced tumor survival by overactive Akt signaling, which is exacerbated by PTEN loss, is common in enzalutamide-resistant tumors (63).

Antiandrogens bind the AR LBD to inhibit full-length AR and thus have no effects on constitutively active AR splice variants, such as ARv567es and V7, which do not express a functional LBD. Expression of ARv567es and V7 is repressed by androgen and increased in the absence of androgen, thereby mediating transcription of target genes when the full-length AR is not transactivated by androgen under castrate conditions. ARv567es is predominantly nuclear (64), whereas the cellular localization of V7 is more variable. In malignant prostate tissue, V7 is predominantly localized in the cytoplasm but nuclear in CRPC (65). Due to the nuclear localization of ARv567es and V7 in CRPC, it has been proposed that they do not mediate non-genomic signaling. In support of this concept, expression of ARv567es results in decreased levels of IGF-1R mRNA and other genes (64) known to be upregulated by non-genomic signaling (66). Other splice variants have been discovered without any genomic function and are likely to carry out non-genomic actions, mainly AR8 and AR23. AR8 encodes solely the AR NTD and a unique 33-amino acid sequence at the C-terminus (67). AR8 has no transcriptional activity and localizes on the cell membrane, possibly by palmitoylation of one or two cysteines in the unique C-terminal sequence (67). Supporting a role in non-genomic signaling, AR8 interacts with full-length AR and EGFR and associates with Src upon EGF stimulation (67). AR8 is endogenously expressed in several CRPC cell lines (C4-2B, 22Rv1, and CWR-R1) and enhances androgen-independent growth (67). AR23 is a cytosolic AR variant carrying a 23-amino acid insertion between the zinc fingers of the AR DBD (68). Interestingly, AR23 is able to potentiate transcription mediated by full-length AR and influence transactivation of NF-kB in reporter gene assays, even though upon ligand binding it is unable to enter the nucleus and regulate gene expression (68).

### Novel AR Therapies

**Figure 2:**
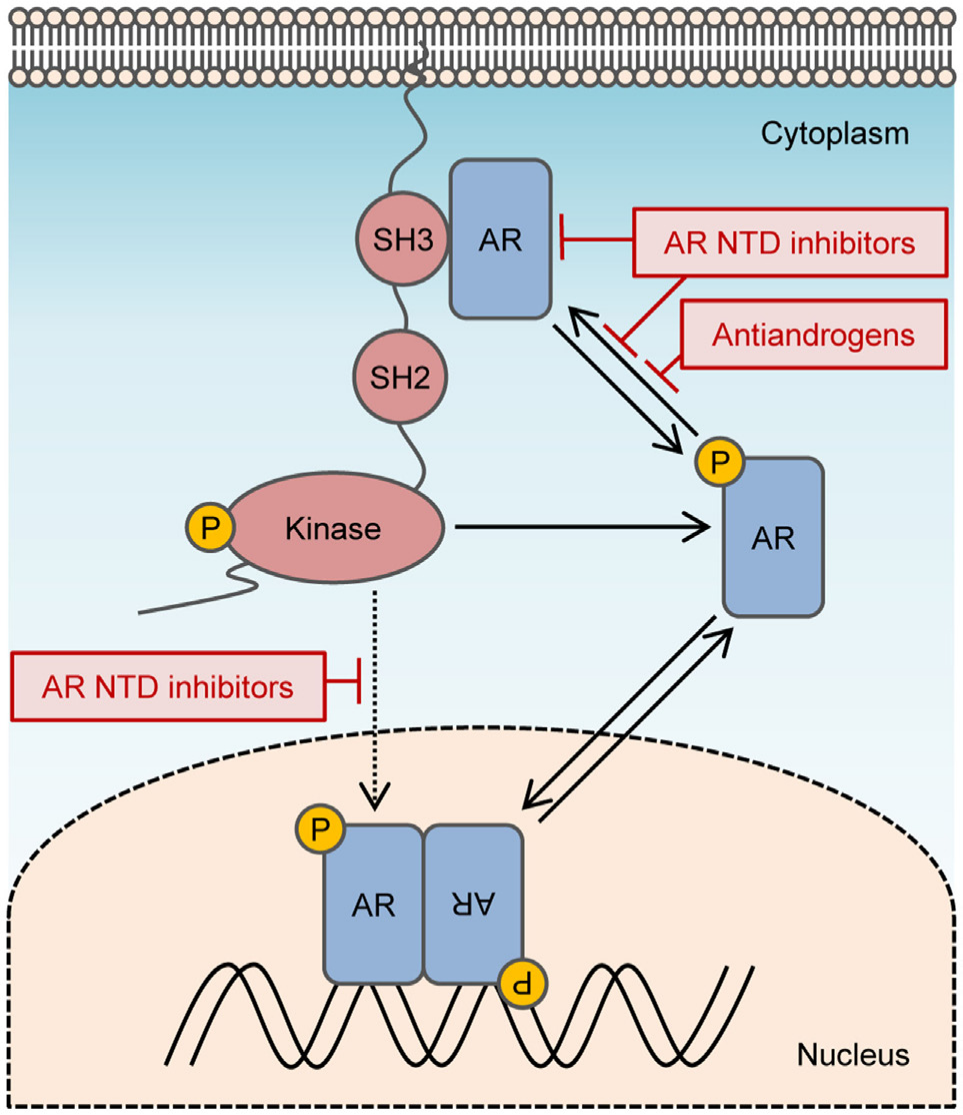
**Targeting non-genomic actions of androgen receptor (AR)**. Non-genomic signaling of AR is activated by interactions between the polyproline domain of the AR N-terminal domain (NTD) and Src homology domain 3 of Src. Activated Src can enhance the transactivation of AR directly by phosphorylating AR Y534 or indirectly by stimulating alternate kinase pathways that modulate AR activity. AR NTD inhibitors may prevent non-genomic signals from AR by blocking Src interaction with the AR NTD and can block both ligand-dependent and ligand-independent transactivation of AR.

Inhibitors targeting the AR NTD might be promising candidates for blocking non-genomic AR signals that enhance tumor growth or survival. To date, two families of compounds have been discovered that bind to the AF1 region of the AR NTD and inhibit transactivation. These are sintokamide A and EPI compounds (69, 70). Both of these agents demonstrate promising results for inducing the regression of enzalutamide-resistant CRPC xenografts in mice (70, 71). Sintokamide A and EPI have different mechanisms of action by binding unique sites on AF1. Nuclear magnetic resonance spectroscopy analyses demonstrate that EPI-001 binds to a specific pocket formed by Tau5 of AR NTD with contact to three regions, residues 353–364, 397–407, and 433–466 (72), that overlaps with the residues 371–381 containing the polyproline sequence required to interact with Src. EPI blocks protein–protein interactions on the AR NTD, between AF1 and CREB-binding protein and TFIIF of the transcription preinitiation complex, and between full-length AR and STAT3, which binds within residues 234–558 of the AR NTD (7, 69, 70). EPI-506 is the first AR NTD antagonist to be tested in clinical trials for metastatic CRPC (NCT02606123). A clinically important advantage to AR NTD inhibitors is that they do not depend on the presence of AR LBD for inhibiting the transcriptional activity of full-length AR as well as AR splice variants. Non-genomic signals from full-length AR, or possibly membrane-associated or cytosolic AR splice variants, such as AR8 or AR23, may be prevented by AR NTD inhibitors that block interaction with Src (Figure 2). Another potential strategy to prevent non-genomic action of AR is to degrade the AR protein. However, all AR degraders to date bind the AR LBD or other non-AR targets. AR degraders that have being tested in clinical trials for CRPC include galeterone (TOK-001) and niclosamide (73–75). The randomized phase III clinical study (NCT02438007) for galeterone was halted recently due to the unlikelihood of meeting its primary endpoint, whereas niclosamide currently entered phase I trials as a combination treatment with enzalutamide (NCT02532114).

### Combined Inhibition of AR and Non-Genomic Substrates

Along with androgen deprivation therapy, non-genomic AR signaling substrates may need pharmacological inhibition to provide maximum benefit to CRPC patients. Several inhibitors of Src family kinases have been tested in a clinical setting for prostate cancer, notably inhibitors that target kinase activity, dasatinib (BMSS-354825) and saracatinib (AZD0530); and KX2-391, a peptidomimetic that blocks the substrate binding site of Src. Clinical studies indicate that targeting Src or inhibiting activated downstream kinase pathways in isolation is ineffective for CRPC (37, 76–79). Likewise, inhibitors of PI3K, Akt, or mTOR have also demonstrated limited use in clinical practice as single agents (80, 81). This is probably because targeting non-genomic AR signals does not protect against ligand-dependent activation of AR and transcription of AR target genes. Inhibition of both non-genomic and genomic pathways of AR may be necessary to eradicate tumor dependency of AR. Concurrent inhibition of AR and non-genomic AR components may prove useful for prostate cancer patients with progression after primary therapy. Many of these strategies are currently under investigation and show promising results in preclinical models of CRPC (82–85).

## Author Contributions

JL and MS both provided substantial contributions to the conception of the work and interpretation of literature. JL provided the first draft and MS revised the work for important intellectual content. JL and MS both approved the version submitted. JL and MS agreed to be accountable for all aspects of the work in ensuring that questions related to the accuracy or integrity of any part of the work are appropriately investigated and resolved.

## Conflict of Interest Statement

MS is a director and officer of ESSA Pharma Inc. that has licensed EPI and sintokamide technologies. MS has equity and receives consulting fees from ESSA Pharma Inc. JL declares that the research was conducted in the absence of any commercial or financial relationships that could be construed as a potential conflict of interest.
